# Spatial Epidemiologic Analysis and Risk Factors for Nontuberculous Mycobacteria Infections, Missouri, USA, 2008–2019

**DOI:** 10.3201/eid2908.230378

**Published:** 2023-08

**Authors:** Carlos Mejia-Chew, Miguel A. Chavez, Min Lian, Angela McKee, Leighton Garrett, Thomas C. Bailey, Andrej Spec, Mansi Agarwal, George Turabelidze

**Affiliations:** Washington University School of Medicine, St. Louis, Missouri, USA (C. Mejia-Chew, M.A. Chavez, M. Lian, T.C. Bailey, A. Spec, M. Agarwal);; Missouri Department of Health and Senior Services. Jefferson City, Missouri, USA (A. McKee, L. Garrett);; Missouri Department of Health and Senior Services. St. Louis (G. Turabelidze)

**Keywords:** nontuberculous mycobacteria, NTM, tuberculosis and other mycobacteria, bacteria, airborne infections, epidemiology, geographic information systems, GIS, Missouri, United States

## Abstract

Nontuberculous mycobacteria (NTM) infections are caused by environmental exposure. We describe spatial distribution of NTM infections and associations with sociodemographic factors and flooding in Missouri, USA. Our retrospective analysis of mycobacterial cultures reported to the Missouri Department of Health and Social Services surveillance system during January 1, 2008–December 31, 2019, detected geographic clusters of infection. Multilevel Poisson regression quantified small-area geographic variations and identified characteristics associated with risk for infection. Median county-level NTM infection rate was 66.33 (interquartile range 51–91)/100,000 persons. Risk of clustering was significantly higher in rural areas (rate ratio 2.82, 95% CI 1.90–4.19) and in counties with >5 floodings per year versus no flooding (rate ratio 1.38, 95% CI 1.26–1.52). Higher risk for NTM infection was associated with older age, rurality, and more flooding. Clinicians and public health professionals should be aware of increased risk for NTM infections, especially in similar environments.

Nontuberculous mycobacteria (NTM) are ubiquitous, environmental, opportunistic microorganisms. Most NTM infections are acquired by inhalation, microaspiration, or direct inoculation (*1*). A recent meta-analysis using data from cultured samples found the global rate of change in NTM disease showed an increase of 4.1% per 100,000 persons per year (*2*). In the United States, 2 recent studies using medical claims data reported an increase in incidence of NTM disease by 7.5% per year (*3*,*4*). One study found the lowest prevalence of NTM pulmonary disease (NTM-PD) among Medicare beneficiaries in the US Midwest and classified the region at low risk for NTM-PD clustering (*4*). However, another study examined 5 US states, including Missouri, a state located in the midwestern United States with an estimated population of 6 million (*5*), reported an annual increase of 9.9% over a 6-year study period. Missouri showed the most yearly variability in NTM prevalence rates; some rates were almost 3 times lower than in the other states studied (*6*). 

Geographic differences in distribution of NTM species likely related to local climate factors or population density variations have been observed worldwide (*2*,*7**–**9*). One study identified specific watersheds near densely populated areas in Colorado, USA, associated with increased risk for clustering of slow-growing NTM infections (*10*). A study in Queensland, Australia, found higher risk for *Mycobacterium intracellulare* infection associated with shallower soil depth and *M. kansasii* with higher soil density (*11*). Another study in Queensland examined the effects of climatic factors on infection trends and found slow-growing NTM incidence increased after a lag period of several months after heavy rainfall, possibly because of the time required for rain to disperse and transport bioaerosols (*12*). However, a prolonged lag period between exposure and disease manifestation is thought to be the norm in NTM disease, so quantifying the effects of individual climatic events is difficult. 

Furthermore, extreme weather events such as heavy rainfall, flooding, and drought likely influence the prevalence of additional environmental organisms (*13*). One study reported increased cases of NTM infections associated with higher numbers of hurricanes affecting the state of Florida (*14*). The proportion of dry to wet areas in Missouri is similar to those of other states considered highly burdened with NTM-PD (*4*). Also, similar to other midwestern states, natural disasters such as tornadoes and floods are common, and earthquakes occur periodically (*15*). Flooding is one of the deadliest severe weather hazards in Missouri because the state is traversed by the Mississippi, Missouri, and White Rivers and their basins (*15*). Using Missouri Department of Health and Senior Services (MDHSS) NTM surveillance data, we aimed to identify spatial clusters of NTM infections and correlate them with sociodemographic factors and seasonal flooding patterns to identify factors associated with higher rates of infection. Washington University (St. Louis, MO, USA) and MDHSS institutional review boards approved this study. 

## Methods

### Patient Population

NTM infection is a reportable condition in Missouri. The surveillance database contains patient sex assigned at birth, date of birth, residential address or postal (ZIP) code, specimen source of the culture, collection date, NTM species isolated, and date on which positive result was reported. We extracted all reports of NTM infections from the MDHSS communicable disease surveillance database collected during January 1, 2008–December 31, 2019. 

### Definitions

To be included, cases needed to have >1 mycobacterial culture positive for an NTM species and residential address or postal code for the sample donor. We excluded duplicate cultures and those positive for *M. gordonae*, given its low pathogenicity (*16*). We defined extrapulmonary NTM infection as a positive culture from a nonrespiratory specimen. For NTM-PD, we applied the microbiologic diagnostic criteria recommended by current guidelines developed by leading international respiratory medicine and infectious diseases societies for defining a case: 2 positive sputum cultures with the same NTM species or a single positive culture obtained through bronchoscopy (*17*). For subanalyses, we grouped NTM into slow-growing and rapid-growing species (*18*). We based annual incidence rates on the number of persons in a calendar year positive for an NTM isolate (infection rate) or fulfilling disease criteria (pulmonary and extrapulmonary disease rates) divided by the population of Missouri in the year of sampling, according to 2010–2019 US Census data (*5*). We calculated index rates using only the first NTM-positive culture from each individual participant. 

### Descriptive Analysis

For categorical variables, we summarized descriptive statistics for persons with NTM cultures using sample proportions. For continuous variables, we used sample medians and interquartile ranges (IQRs).

### Spatial Statistical and Multilevel Analyses

Using spatial and space-time scan statistics (*19*,*20*) based on census tract–level coordinates and counts (i.e., centroids of census tracts, NTM cases, background population sizes), we applied a Poisson model to detect geographic hotspots of higher-than-expected NTM infections. We defined hotspots as areas in which NTM infection rates were significantly higher than the statewide average and elsewhere in Missouri. To infer the statistical significance of each potential cluster, we applied a circular window with a varied radius (<50% of the total at-risk population in the study area) to scan the study area and generate 999 Monte Carlo permutation datasets for computing the statistics. 

Because we considered the general population at risk, a multilevel framework (individual patients nested in their residential counties) was necessary to control bias from potential correlations of patients residing in the same county. We performed multilevel Poisson regression analysis to generate the predicted county-level incidence rates of NTM infection, quantify the small-area geographic variation in NTM infections, and identify neighborhood characteristics associated with NTM infections. The terms small-area and neighborhood refer to census tracts in cluster analyses and counties in multivariate multilevel Poisson regression. To remove potential bias from small populations in some counties when estimating county-level NTM infection rates, we used multilevel modeling–based prediction (adjusting for age, sex, and race and ethnicity) instead of observed values to report the smoothed rate. For multilevel Poisson regression analysis of predicted county-level incidence rates, we adjusted the model for demographics only to generate smoothed small-area incidence rates. To quantify small-area geographic variations in NTM infections and identify neighborhood characteristics associated with NTM infections, we fit multilevel Poisson regression to a single multivariate model to estimate county-level variation in NTM incidence (random effect measured by median rate ratio [MRR]) and potential associations of county-level factors (fixed effects measured by rate ratio [RR]). 

We further integrated the NTM county-population dataset with neighborhood contextual measures, including dates of flooding events in specific counties during 2008–2019, rural–urban context (defined as rural, urban, or metropolitan using the rural–urban continuum area code from the US Department of Agriculture; https://www.ers.usda.gov/data-products/rural-urban-continuum-codes), county-level percentage of population below federal poverty line, and county-level percentage of non-Hispanic Black population. We did not include other minorities, which represented <5% of the state population, in the analyses (*5*). We also used an adjusted multivariate multilevel model for individual-level demographics (age, sex, and race and ethnicity). We reported MRR, a measure of geographic heterogeneity with a value >1 (*21*), because it reflects the average difference between a pair of counties randomly selected from the study area, with a higher value indicating more small-area variation. 

We analyzed geographic clusters by using SaTScan software version 9.7 (https://www.satscan.org) and managed datasets and performed multilevel modeling in SAS version 9.4 (SAS Institute Inc., https://www.sas.com). We visualized identified census tracts included in the significant clusters and predicted/smoothed county-level incidence rates by using the ArcGIS software package version 10.6.1 (ESRI https://www.esri.com). 

## Results 

### Cohort Characteristics

We identified 14,203 mycobacterial cultures reported to MDHSS during the study period, of which 10,996 met the inclusion criteria. After excluding 77 duplicates and 1,450 *M. gordonae* isolates, we included 9,469 culture-positive samples from 5,288 persons in the analyses. Median age of persons with NTM infection was 67 years (IQR 54–76 years); 52.1% were White and 52.7% female. A total of 3,292 (62%) persons provided respiratory cultures and 481 (9.1%) extrapulmonary cultures; culture source was unknown for 1,515 (28.6%). Smoothed median rate of NTM infection was 68.04 (IQR 59.65–81.12)/100,000 persons for the study period, and compared with the 2008 baseline, yearly rate of infections had increased 5.7% by 2010 and 12.2% by 2019. 

The 5 most frequently isolated NTM species were *M. avium* (60.1%), *M. fortuitum* (8.3%), *M. abscessus* (6.5%), *M. chelonae* (5.6%), and *M. kansasii* (3.8%). Among isolates, 72% were slow-growing NTM; median time to positivity from culture collection was 20 days (IQR 13–30 days) (Table 1). The proportion of new isolates per NTM species remained stable during 2008–2019, except for *M. avium*, which exhibited a positive but not statistically significant increase (19.6%, p = 0.067) (Appendix Figure). 

**Table 1:** Characteristics of 5,288 patients with NTM infections, Missouri, USA, 2008–2019*

Characteristics	Value†
Demographics	
Median age, y (IQR)	67 (54–76)
Sex‡	
F	2,785 (52.7)
M	2,498 (47.2)
Not recorded	5 (0.1)
Race/ethnicity	
Non-Hispanic White	2,753 (52.1)
Non-Hispanic Black	459 (8.7)
Asian	91 (1.7)
Other/unknown	1,985 (37.5)
NTM characteristics	
Median NTM rate (IQR)†	68.04 (59.65–81.12)
Slow-growing mycobacteria	3,806 (72.0)
* Mycobacterium avium*	4,752 (60.13)
* M. kansasii*	305 (3.86)
Rapid-growing mycobacteria	1,482 (28.0)
* M. fortuitum*	660 (8.35)
* M. chelonae*	230 (6.69)
* M. abscessus*	196 (5.7)
Median time to culture positivity, d (IQR), n = 4,956	20 (13–30)

*Values are no. (%) patients except as indicated. NTM, nontuberculous mycobacteria; IQR, interquartile range. †Over study period, per 100,000 population.

Using the standardized population of Missouri, we estimated an age-adjusted rate of NMT infection of 84.80/100,000 persons (82.28 for men, 87.09 for women) and 29.87/100,000 persons for NTM-PD (25.68 for men, 33.85 for women). By type of NTM, age-adjusted rates were 24.04/100,000 persons for rapid-growing infections and 60.76/100,000 persons for slow-growing infections (Appendix Table 1). 

### Pulmonary and Extrapulmonary Nontuberculous Mycobacterial Disease

Among all NTMs detected in respiratory specimens, 1,875 (56.9%) fulfilled the microbiologic diagnostic criteria for NTM-PD. Patients with NTM-PD were more commonly female (58% vs. 43.5%; p <0.001), older (median age 70 vs. 59 years; p <0.001), and infected with slow-growing NTM (83.9% vs. 48.6%) compared with those with extrapulmonary disease (Appendix Table 2). Among the 669 (35.7%) patients with NTM-PD who provided >1 respiratory sample with the same NTM species isolated, the predominant NTM species was *M. avium* (81.2%), followed by *M. kansasii* (4.19%) and *M. abscessus* (3.9%). Among the 481 (9.1%) patients with extrapulmonary infection, the most commonly isolated NTM species were *M. avium* (37.9%), *M. fortuitum* (17.2%), *M. chelonae* (14.9%), and *M. abscessus* (8.1%). 

### Geographic Variation and Clustering of NTM Infections 

**Figure 1:**
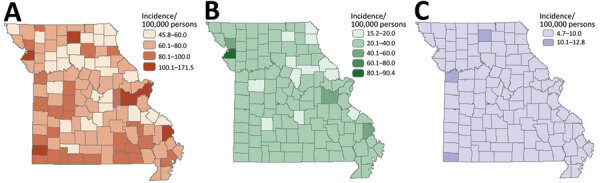
Smoothed county-level incidence rates of nontuberculous mycobacterial (NTM) infections, by infection site, Missouri, USA, 2008–2019. A) All NTM; B) pulmonary NTM; C) extrapulmonary NTM.

**Figure 2:**
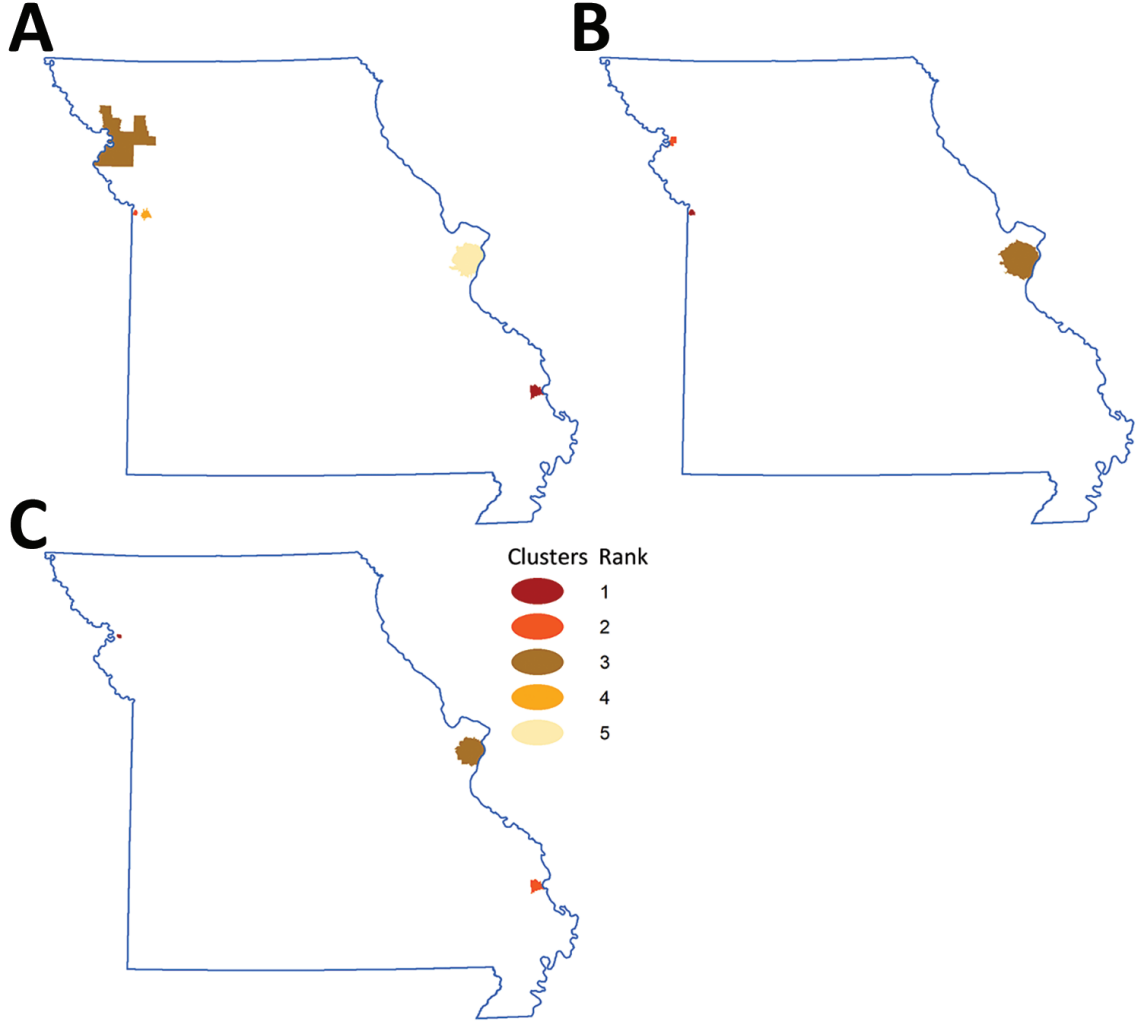
Geographic hotspots of nontuberculous mycobacteria (NTM) infection, by NTM type, Missouri, USA, 2008–2019. Colors indicate rank order, based on relative risk point estimates. A) For all NTM infections, relative risk was 3.62 for rank 1, 2.45 for rank 2, 2.19 for rank 3, 1.66 for rank 4, and 1.53 for rank 5. B) For rapid-growing NTM, relative risk was 3.84 for rank 1, 2.69 for rank 2, and 1.99 for rank 3. C) For slow-growing NTM, relative risk was 5.42 for rank 1, 4.52 for rank 2, and 1.42 for rank 3.

We excluded data from the 4.95% of geocoded locations outside the state of Missouri from further analyses. During the study period, the counties with the highest incidence were all rural: Buchanan (171.46/100,000 persons), Cape Girardeau (134.81/100,000 persons), and Sullivan (121.1/100,000 persons); those findings were mainly driven by high NTM-PD incidence rates in Cape Girardeau (90.45/100,000 persons) and Buchanan County (58.9/100,000 persons). By comparison, incidence of NTM infections in the 2 most populous metropolitan areas were 111.1/100,000 persons for St. Louis County, in which St. Louis is located, and 95.91/100,000 persons for Jackson County, in which Kansas City is located. The 3 counties with the highest incidence rates of extrapulmonary NTM cases were Sullivan (12.8/100,000 persons), McDonald (11.8/100,000 persons), and Jackson (10.6/100,000 persons) (Figure 1). Using spatial and space-time scan statistics across the state, we identified hotspots of significantly higher-than-expected NTM infection, NTM-PD, and extrapulmonary disease (Figure 2). Persons living in Cape Girardeau County were 3.62 times more likely to have any NTM infection and 4.52 times more likely to have slow-growing NTM infection than persons living elsewhere in Missouri. 

### Multilevel Analysis of Contextual and Individual Characteristics of NTM Infections

Multilevel Poisson analysis (Table 2) showed substantial small-area geographic variation in NTM infections across counties (variance 0.32, MRR 1.73; p<0.001). Risk for NTM infections was significantly higher in counties with >5 floods per year than in those with no flooding (RR 1.38, 95% CI 1.26–1.52) but not in counties with the highest poverty rates (highest vs. lowest quartile incomes, RR 0.78, 95% CI 0.54–1.13) or highest percentages of non-Hispanic Black population (highest vs. lowest quartiles, RR 0.84, 95% CI 0.58–1.21). Compared with metropolitan counties, both rural (RR 2.82, 95% CI 1.90–4.19) and urban (RR 2.08, 95% CI 1.53–2.82) counties had higher risks for NTM infection. Compared with persons ≤20 years of age, risk for NTM infection was significantly higher among persons 20–49 years of age (RR 7.23, 95% CI 5.11–10.2) and 50–64 years of age (RR 26.7, 95% CI 18.9–37.7), and even more so among persons ≥65 years of age (RR 76.8, 95% CI 54.6–108.2). Of note, risk of NTM infection was lower among women than men (RR 0.94, 95% CI 0.89–0.99) but higher among non-Hispanic Black persons than among non-Hispanic White persons (RR 2.62, 95% CI 2.31–2.98). 

**Table 2:** Results of multilevel Poisson regression analyses of risk for nontuberculous mycobacterial infection, Missouri, USA, 2008–2019

Variable	Rate ratio (95% CI)
Fixed effects	
County-level flooding	
No flooding	Referent
1–3 times	1.19 (1.11–1.29)
4–5 times	1.29 (1.17–1.43)
>5 times	1.38 (1.26–1.52)
County rural–urban context	
Rural	2.82 (1.90–4.19)
Urban	2.08 (1.53–2.82)
Metro	Referent
County-level poverty levels	
1st quartile (lowest)	Referent
2nd quartile	0.88 (0.62–1.25)
3rd quartile	0.71 (0.49–1.03)
4th quartile (highest)	0.78 (0.54–1.13)
County-level ratio non-Hispanic Black	
1st quartile (lowest)	Referent
2nd quartile	1.17 (0.81–1.69)
3rd quartile	1.09 (0.76–1.56)
4th quartile (highest)	0.84 (0.58–1.21)
Patient age group, y	
<20	Referent
20–49	7.23 (5.11–10.2)
50–64	26.7 (18.9–37.7)
>65	76.8 (54.6–108.2)
Sex	
M	Referent
F	0.94 (0.89–0.99)
Race/ethnicity	
Non-Hispanic White	Referent
Non-Hispanic Black	2.62 (2.31–2.98)
Others*	32.2 (30.3–34.2)
Random effect	
Variance 0.32, p<0.001	1.73

*Asian (91; 1.72%), native American (9; 0.17%), unknown race (1,976; 37%).

## Discussion 

We identified clusters of NTM infections in Missouri associated with sociodemographic factors and flooding. In counties where NTM infection rates were 3–4 times those for the rest of the counties, higher-than-expected rates were associated with older age, rurality, non-Hispanic Black race, male sex, and higher numbers of annual floods. Of note, overall average NTM incidence rate in Missouri was higher in our study than previously reported in large national datasets (*3*,*22*). This discrepancy might be related to differing sources of NTM reporting, because previous studies relied on International Classification of Disease (ICD) codes, which have low sensitivity, to identify NTM cases, not the mandatory laboratory reporting that our study used (*23*). 

In keeping with the known epidemiology of NTM infections, most cultures in our study came from respiratory sources (*17*). NTM-PD rates approximated those previously described in epidemiologic studies from large administrative healthcare sources (*3*,*4*,*6*). More than half the persons in our study were ≥65 years of age and female, both factors significantly associated with NTM-PD. Of note, on the basis of the multilevel Poisson analysis, women had a slightly lower risk of NTM infection than men. Like increased NTM infections reported worldwide (*2*), *M. avium* was the most common NTM species in both pulmonary and extrapulmonary infections and the only NTM species that exhibited an increasing incidence over time. Reasons for this reported increase are likely multifactorial and include better mycobacterial diagnostic tools, increased NTM disease awareness, and extreme weather events disrupting the NTM ecologic niche (*13*). However, unlike in other locations worldwide, the number of *M. abscessus* infections reported in Missouri during the study period remained stable. 

We found that counties in Missouri with >5 flooding events per year had a 38% higher rate of NTM infections than those without flooding. A study conducted in Florida found higher numbers of hurricanes, which can lead to flooding, associated with higher numbers of NTM infections (*14*). Those findings support the hypothesis that trends in flooding events may correlate with NTM infection rates, possibly because disruptions in the ecosystem of environmental mycobacteria from extreme weather events could increase human exposure and risk for potential infection. 

Our study was limited by its retrospective design and use of mandatory laboratory reporting data. Lack of clinical data did not enable us to differentiate between disease and colonization; for this reason, we used the term NTM infection throughout the text and used NTM-PD only when patients fulfilled microbiologic diagnostic criteria for NTM disease. In addition, the MDHSS NTM surveillance database was not routinely queried for inconsistencies; hence, incomplete data on key variables could have introduced bias. However, except for 37% missing or unknown entries for race, missingness was <5% for key variables and unlikely to have biased analyses. Furthermore, other environmental factors identified in previous studies (*11*,*12*) could have influenced NTM infection rates, but we focused on a factor, flooding, that had not been studied before. 

In conclusion, we identified increasing rates of NTM infection over time. NTM infection clustering in Missouri was associated with older age, rurality, and higher rates of annual flooding events. Further investigation is warranted to determine the degree to which extreme weather events contribute to the increasing incidence and prevalence of NTM-PD worldwide. In addition, clinicians and public health professionals should be aware of the increased risk for NTM infections, especially in locations with environments similar to those described here. 

Appendix Additional information on factors associated with incidence of nontuberculous mycobacteria infections in Missouri, USA, 2008–2019.
