# Supplementary material for: Spatial Epidemiologic Analysis and Risk Factors for Nontuberculous Mycobacteria Infections, Missouri, USA, 2008–2019
Source: Emerg Infect Dis. 2023 Aug;29(8):1540–6. doi: 10.3201/eid2908.230378 (PMC10370856; doi:10.3201/eid2908.230378)
Supplement: Appendix — Additional information on factors associated with incidence of nontuberculous mycobacteria infections in Missouri, USA, 2008–2019. [file 23-0378-Techapp-s1.pdf]

Article DOI: http://doi.org/10.3201/eid2908.230378

EID cannot ensure accessibility for supplementary materials supplied by authors. Readers who have difficulty accessing supplementary content should contact the authors for assistance.

Spatial Epidemiologic Analysis and Risk Factors for Nontuberculous Mycobacteria Infections, Missouri, USA, 2008–2019

Appendix

Appendix Table 1. Age-adjusted rates of NTM infection per 100,000 persons in Missouri, United States, 2008–2019*

Type of NTM infection	Total population	M	F
All NTM cases	84.80	82.28	87.09
Pulmonary disease	29.87	25.68	33.85
Extrapulmonary disease	7.85	9.05	6.69
Rapid-growing mycobacteria	24.04	25.74	22.38
Slow-growing mycobacteria	60.76	56.54	64.71

*NTM, nontuberculous mycobacteria.

Appendix Table 2. Characteristics of persons with NTM in Missouri, United States, 2008–2019*

Characteristics	Pulmonary n = 1,875 (%)	Extrapulmonary n = 481 (%)	p value
Demographics			
Median age in years (IQR)	70 (59–78)	59 (45–71)	<.001
Female sex†	1088 (58)	209 (43.5)	<.001
Race			
Non-Hispanic White	1082 (57.7)	276 (57.4)	<.001
Non-Hispanic Black	89 (4.7)	63 (13.1)	
Asian	26 (1.4)	1 (0.2)	
Other/unknown race	678 (36.2)	141 (29.3)	
NTM characteristics			
Median NTM rate per 100,000 population (IQR)‡	27.7 (23.15–31.13)	6.1 (5.6–7.37)	NA
Rapid-growing mycobacteria	302 (16.1%)	247 (51.4%)	<.001
Slow-growing mycobacteria	1573 (83.9%)	234 (48.6%)	<.001
Median time to culture positivity in days (IQR)	18 (13–28), n = 1,789	19 (12–30), n = 457	<.001

*IQR, interquartile range; NA, not available; NTM, nontuberculous mycobacteria.
†For 2 (0.1%) persons sex was not recorded.
‡Per study period.

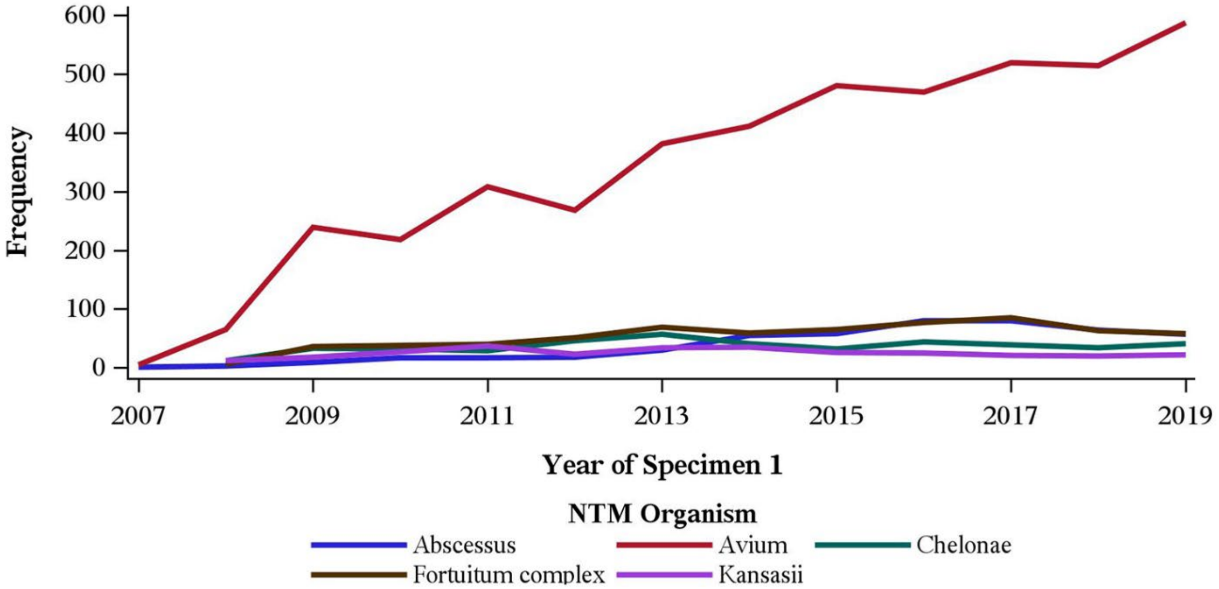

Appendix Figure. Incidence trend for the most common nontuberculous mycobacterial infections in Missouri, USA, 2008–2019. Number of nontuberculous mycobacteria infections reported yearly for the 5 most common nontuberculous mycobacteria species isolated: 2008 (100, 1.99%), 2009 (290, 5.78%), 2010 (286, 5.7%), 2011 (378, 7.53%), 2012 (385, 7.67%), 2013 (461, 9.19%), 2014 (459, 9.15%), 2015 (489, 9.75%), 2016 (488, 9.73%), 2017 (538, 10.72%), 2018 (529, 10.54%), 2019 (614, 12.24%).
